# A systematic review and meta-analysis of motivational climate and youth sport outcomes: examining a hierarchical effects model

**DOI:** 10.3389/fpsyg.2025.1716745

**Published:** 2026-01-08

**Authors:** Yimeng Gu, Long Cheng

**Affiliations:** School of Wushu, Henan University, Kaifeng, China

**Keywords:** meta-analysis, motivational climate, adolescents, physical activity, physical education class

## Abstract

**Objective:**

Although the beneficial impact of motivational climate on adolescent sport participation is well-established, key questions remain unresolved regarding its hierarchical mechanism and the relative efficacy across climate types. This study applies a meta-analytic approach to reveal the deep structural mechanism of motivational climate to guide theoretical improvement and provide empirical support for optimal implementation in physical education.

**Methods:**

Following PRISMA 2020 guidelines, we systematically searched the Web of Science and CNKI databases for studies published between 2015 and 2025. The methodological quality of the included studies was assessed using JBI critical appraisal tools. A total of 177 studies, yielding 334 independent effect sizes, were included in the final analysis. A random-effects model was used to conduct the main meta-analysis, moderator analyses, and publication bias tests.

**Results:**

The meta-analysis revealed a moderate overall positive association between motivational climate and youth outcomes (*r* = 0.31), indicating that the social environment is a meaningful pedagogical lever for enhancing student experiences. However, the substantial heterogeneity (*I*^2^ = 98.45%) foreshadows that this influence is not uniform, but rather varies structurally depending on whether the outcome is psychological or behavioral. Moderator analysis provided strong evidence for a hierarchical pathway: the effect was largest on “motivation and cognition” (*r* = 0.42), attenuated for “behavioral & engagement” (*r* = 0.26), and was weakest for “performance and health” (*r* = 0.19). While the “Others” category, comprising emerging climate models, showed a particularly strong association (*r* = 0.43). The moderating effect of publication year was not statistically significant. No significant publication bias was found (Egger's test, *p* = 0.91).

**Conclusion:**

The pattern of association between motivational climate and adolescent outcomes is consistent with a psychological-to-behavioral hierarchical model. This underscores motivational climate as a key “psychological architect” that influences longer-term behavior and performance indirectly through internal psychological states. The study highlights aspects of this motivational climate by not only demonstrating its positive influence, but also unmasking its structural pathways and offering us promising climate models with some educational significance.

**Systematic review registration:**

https://www.crd.york.ac.uk/PROSPERO/view/CRD420251155621, identifier: CRD420251155621.

## 1 Introduction

Promoting ongoing engagement of young people in physical activity is an important public health objective, and the motivational atmosphere provided by coaches and teachers is thought to be an important factor of success (Ntoumanis, 2001). Motivational climate is defined as the social-psychological environment formed by significant others such as teachers, coaches, and peers. It exerts a strong motivational influence on individuals through situational goals, feedback behaviors, and interpersonal styles of interaction (Duda and Appleton, 2016).

Based on achievement goal theory and self-determination theory, motivational climate is primarily categorized into “task-oriented/mastery climate” and “performance-oriented/outcome climate,” and the emerging “empowering climate” in recent years (Duda and Ntoumanis, 2005). These theories all emphasize the mechanisms by which people's motivational regulation, self-efficacy, tendency to attribute, and wellbeing are influenced by their surrounding environment. Empirical evidence shows that mastery or empowering climates are often associated with higher intrinsic motivation, long-term physical activity practice, lower levels of anxiety, and higher levels of psychological wellbeing (Reinboth and Duda, 2004). In contrast, performance climates are possibly associated with harmful results such as stress, burnout, and antisocial behaviors (Bartholomew et al., 2011). However, research evidence is not entirely united to this day yet. This lack of consensus creates confusion for practitioners and complicates the development of evidence-based coaching and teaching interventions. This gap is particularly critical in school contexts, where recent syntheses emphasize that structured, evidence-based climates are pivotal for fostering socio-emotional intelligence and positive student development (Baena Extremera et al., 2025). However, without a clear understanding of the underlying mechanisms, translating these goals into practice remains difficult. There is some research that reports the coexistence of both task-oriented and performance-oriented climates under different cultural or teaching contexts (Murcia et al., 2007), and some intervention research reflects that short-term effects of motivational climate intervention may not become stable in a long run (Sevil-Serrano et al., 2020).

In order to address these inconsistencies, and offer a more in-depth illumination of the evidence, this meta-analysis seeks to systematically unpack the structural pathways of motivational climate's effects on youth sport outcomes. However, a simple correlation is insufficient to understand the mechanism. Drawing from Self-Determination Theory (Ryan and Deci, 2000), we propose an a priori hierarchical model of influence. Theoretically, motivational climate acts as an external social-contextual factor that does not directly dictate behavior; rather, it first impacts an individual's internal psychological states (e.g., need satisfaction, motivation). These internalized states then serve as the proximal drivers for external behavioral engagement and subsequent performance. Therefore, we hypothesize a “diminishing hierarchy” of effects: the association between climate and outcomes will be strongest at the internal psychological level and systematically attenuate as the outcomes become more distal (i.e., behavioral and performance indicators). This study aims to empirically test this theoretical hierarchy through meta-analytic evidence. Specifically, we will ask three questions: First, does the climate effect yield a hierarchical pathway stemming from internal psychological states to external behaviors? Second, is there an establishment of a robust efficacy hierarchy of different climate types? Third, what temporal pattern exists for reported effects across the past decade? When answering these questions, this study will move beyond simply affirming the climate's positive impact, to explicating is core processes and setting a clear evidence base for future research and practice.

## 2 Data source and methods

### 2.1 Eligibility criteria and study selection

To ensure the rigorous selection of studies, specific inclusion and exclusion criteria were established. The inclusion criteria were as follows: (1) The study must explicitly investigate “motivational climate” as a socio-psychological environment; (2) The research must be grounded in or related to theoretical frameworks such as achievement goal theory, self-determination theory, or caring climate theory; (3) The motivational climate must be constructed by “others,” including coaches, teachers, parents, peers, or teams as a whole; (4) The study must be conducted within the domain of physical activity or physical education; (5) The study must measure and report at least one quantifiable outcome variable, which can be categorized into motivation, behavior, emotional, or cognitive responses, and the data must be available; (6) The study must be a quantitative empirical study, such as an experimental, cross-sectional, or longitudinal study.

The exclusion criteria were as follows: (1) The study does not focus on motivational climate or defines “climate” in ways inconsistent with the construct examined in this research; (2) The research setting is not within physical activity or physical education contexts; (3) The study population does not consist of adolescents; (4) The study is purely qualitative, a literature review, a theoretical or opinion article, or lacks sufficient quantitative data (e.g., means, standard deviations, correlation coefficients) to calculate effect sizes; (5) The article is not peer-reviewed, such as conference abstracts or theses; (6) The language of the publication is neither English nor Chinese, or the full text is inaccessible for evaluation.

Based on these criteria, the parameters for this review were structured according to the PICOS model (Population, Intervention, Comparison, Outcomes, and Study Design) as outlined in Table 1.

**Table 1:** PICOS criteria for study inclusion.

**Criteria**	**Description**
Population	Adolescents participating in physical activity or physical education contexts.
Intervention/exposure	Exposure to a motivational climate created by significant others (e.g., coaches, teachers, peers).
Comparison	Different types of motivational climates (e.g., Task-Oriented vs. Performance-Oriented) or implicit comparison to neutral climates.
Outcomes	Any quantifiable outcome related to motivation, cognition, emotion, behavior, sport performance, or health.
Study design	Quantitative empirical studies, including experimental, cross-sectional, or longitudinal designs.

### 2.2 Search strategy

The literature search was independently conducted by the first author and the corresponding author in strict accordance with the PRISMA 2020 Statement (Page et al., 2021). The protocol for this systematic review was registered in the International Prospective Register of PROSPERO, registration number CRD420251155621 (25 September 2025). Relevant studies were retrieved using computer-based searches of the Web of Science and CNKI databases. The search terms included: TS = (“motivational climate” OR “caring climate” OR “autonomy support”) AND (“physical education” OR “sport” OR “exercise”) AND (“adolescent” OR “youth” OR “student”). The search covered literature published between 2015 and 2025 and was systematically carried out by two researchers using a combination of subject terms and free-text keywords. Detailed search formulas in Appendix A.

### 2.3 Literature selection

After initial screening based on the predefined inclusion and exclusion criteria, the first author and the corresponding author independently extracted data using a standardized Data Extraction Form, with all entries cross-checked. Disagreements were resolved through discussion or consultation with relevant experts. The extracted information included basic publication details (title, authors, publication year), study population, research setting, study type, core constructs, control group information, outcome variables, study design, and data availability.

### 2.4 Risk of bias assessment

Two independent reviewers assessed the methodological quality and risk of bias of each study included in this review utilizing the relevant Joanna Briggs Institute (JBI) critical appraisal checklist based on study design. Disagreement regarding assignment of the assessors' ratings was resolved by consensus. Based on percentage of criteria met, studies were assigned a low (≥70%), moderate (50–69%), or high (<50%) risk of bias rating. The risk of bias rating was intended to be used as a potential moderator in subgroup analysis to examine heterogeneity.

### 2.5 Statistical analysis

Selected publications on the impact of motivational climate on adolescents in physical activity contexts were subjected to a number of statistical procedures using R software. Such procedures included main effect analysis, heterogeneity testing, moderator effect analysis (subgroup analysis using independent and dependent variables and meta-regression), main effect analysis and heterogeneity testing. Random-effects meta-analysis was performed first to obtain pooled average effect size and corresponding confidence interval for judging overall strength of association between motivational climate and disparate outcomes. Thereafter, an assessment of effect sizes' consistency was performed using *I*^2^ statistic. Heterogeneity testing was performed if *I*^2^ was too large. It involved meta-regression using publication year as a covariate and subgroup analysis using independent and dependent variables to investigate possible origins of heterogeneity. Finally, robustness of results was also determined using publication bias assessment.

Different statistical measures were reported to present results. For simplicity and to make it possible to conduct subsequent analyses, all reported measures were converted to a common effect size, r. Used was the transformation formula *r* = 0.98 × β + 0.05 × λ (Peterson and Brown, 2005).

We selected this transformation to maximize data inclusion, as a significant proportion of recent high-quality research in this field employs regression or structural equation modeling (SEM). Excluding these studies would result in a substantial loss of data and potential selection bias. Following the recommendations for clarity in descriptive statistical reporting (Ibáñez-López et al., 2024), we ensured that all effect size distributions and forest plots were presented with maximum transparency.

All effect sizes were converted to r before applying Fisher's r-to-z transformation for obtaining increased statistical stability and generating about normally distributed z values with the formula Z = 0.5 × ln[(1 + r)/(1 – *r*)] (Borenstein et al., 2009). Those z values were subsequently used for calculation of weighted averages.

Common coding was also applied because different studies used separate and distinct labels for independent variables. Concepts originating or having direct associations with self-determination theory were put together in the category of “Autonomy/Empowerment-Supportive Climate.” “Task/Mastery-Oriented Climate” was applied to categorize constructs like “task-involved,” “mastery-oriented,” and “learning-oriented” originating in achievement goal theory. Terms like “ego-involved” and “performance-oriented” were included in the category of “Performance/Ego-Involved Climate.” A category for intervention designs specifically used was also built under the term of “Intervention Study.” Effects of variation of effect sizes through motivational climate theoretical frameworks are made easier by applying this kind of coding method.

## 3 Results

### 3.1 Literature screening process

**Figure 1:**
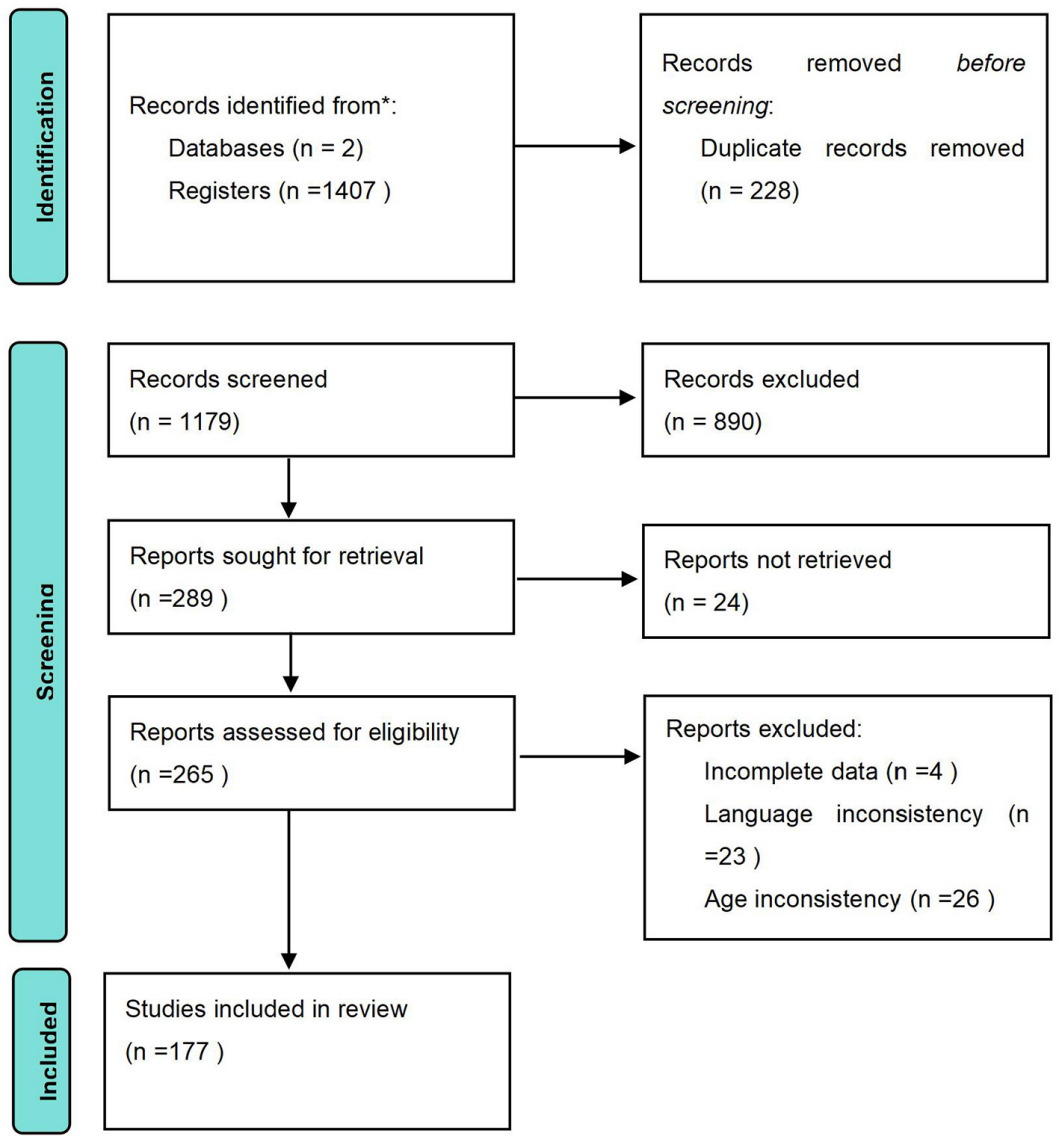
Literature screening process.

The PRISMA flow diagram (Figure 1) summarizes the process for the literature search and selection. After searching the databases, there were a total of 1,407 records. Following the removal of duplicates, 1,179 records were screened by titles and abstracts with 890 excluded. The remaining 289 articles were obtained to assess eligibility. After retrieval, 111 articles were excluded for not meeting the inclusion criteria. The final total included in the meta-analysis was 177 studies (Girard et al., 2019; Guo et al., 2022; Kokkonen et al., 2019; Kim et al., 2025; Kröske, 2017; Castro-Sánchez et al., 2020; Borrego et al., 2021; Zach et al., 2020; Kipp and Weiss, 2015; Brisimis et al., 2022; Jakobsen, 2023; Pop et al., 2022; Ortega et al., 2018; Extremera et al., 2015; Huhtiniemi et al., 2022; Wagnsson et al., 2016; Espinoza Gutiérrez et al., 2024; Stanger et al., 2018; Kavussanu and Ring, 2021; Pineda-Espejel et al., 2021; Coutinho et al., 2024; Chu et al., 2021; Marcelino, 2025; Coudevylle et al., 2015; Pulido et al., 2020; Gaudreau et al., 2016; McLaren et al., 2017; Habeeb et al., 2023; McKay et al., 2025; Morales-Sánchez et al., 2022; Vallerand, 2000; Abós et al., 2017; McLaren et al., 2015; Jaakkola et al., 2016, 2017; Balaguer et al., 2018; Xu et al., 2025; Hogue et al., 2019; Castro-Sánchez et al., 2018; Çaglar et al., 2017; Ulaş et al., 2020; Cruz Feliu et al., 2016; Tristán et al., 2021; Breiger et al., 2015; Hancox et al., 2017; Bengoechea et al., 2017; Danioni et al., 2021; Zhang et al., 2024; Bugten et al., 2025; Morales-Sánchez et al., 2020; Deng et al., 2023; Bolter and Kipp, 2018; Zarrett et al., 2015; Gómez-López et al., 2024; Gustafsson et al., 2016; Alesi et al., 2019; Grastén, 2016; Castro-Sánchez et al., 2019a; Guo et al., 2021; Cheon et al., 2022; Kokkonen et al., 2020; Méndez-Giménez et al., 2018; Gómez-López et al., 2020; Martin and Horn, 2025; Castro-Sánchez et al., 2019c; Sukys et al., 2020; Ettekal et al., 2016; Rodrigues et al., 2024; Marjanović et al., 2019; Lindell-Postigo et al., 2023; Hauser et al., 2024; Cheon et al., 2023; Kim et al., 2016; Ruiz et al., 2017; Castillo-Jiménez et al., 2022; Jang et al., 2020; Wu et al., 2021; Moreno-Rosa et al., 2024; Wang et al., 2021; Ada et al., 2021; Raimundi et al., 2023; Mosqueda et al., 2019; Kipp and Bolter, 2024; Shen, 2015; Morela et al., 2017; Gil-Arias et al., 2020; Castro-Sánchez et al., 2019d; Durdubas et al., 2020; Theodosiou et al., 2021; Flores-Piñero et al., 2024; Elsborg et al., 2023; Cecchini et al., 2019; Buch et al., 2016; Chan et al., 2025; Bortoli et al., 2017; Weeldenburg et al., 2022; Into et al., 2020; Weltevreden et al., 2018; Tamminen et al., 2016; Morela et al., 2021; Álvarez et al., 2019; Rodrigues et al., 2022; Kristensen et al., 2025; Milton et al., 2025; Allami et al., 2022; Mossman et al., 2021; Castillo et al., 2020; Leyton-Román et al., 2020; Lis Velado and Carriedo Cayón, 2019; Gjesdal et al., 2019a,b; Gråstén et al., 2018; Rodrigues et al., 2020; Jiang et al., 2025; Kromerova-Dubinskiene and Sukys, 2020; Nordin-Bates et al., 2016; Castro-Sánchez et al., 2019b; Baena-Extremera et al., 2015; Girard et al., 2022; Battista et al., 2019; Leo et al., 2015; Bortoli et al., 2015; Sánchez Martín et al., 2024; Erickson and Côté, 2016; Zheng et al., 2023; Smith et al., 2016; Davies et al., 2016; Fabra et al., 2021; González et al., 2024; Wang and Zhong, 2025; Saldanha et al., 2020; Granero-Gallegos et al., 2017; Hogue et al., 2017; Wang et al., 2016; Thibodeaux and Winsler, 2022; Bae, 2023; Valero-Valenzuela et al., 2024; Danioni and Barni, 2019; Borghouts et al., 2023; Girard and De Guise, 2024; Silva et al., 2025; Chiva-Bartoll et al., 2018; Rillo-Albert et al., 2021; Nielsen et al., 2024; Park et al., 2019; Zarrett et al., 2021; Pons et al., 2023; Warburton, 2017; Monteiro et al., 2018; Cheon et al., 2019; Gardner et al., 2016; Garcia-Gonzalez et al., 2017; Reverberi et al., 2020; Byrd and Martin, 2016; Baena Extremera et al., 2025; Maldonado et al., 2019; Smith et al., 2015; Zanatta et al., 2018; Gredin et al., 2022; Elbe et al., 2018; Riley et al., 2017; Varga and Révész, 2023; Agans et al., 2018; Gürpinar et al., 2023; Gano-Overway et al., 2017; Chang et al., 2017; McLaren et al., 2024; García-González et al., 2019; González-Hernández et al., 2023; Gómez-López et al., 2019b,c,a; Morela et al., 2019; Curran et al., 2015; Chen et al., 2020; Iwasaki and Fry, 2016; Hogue, 2024; Chamberlin et al., 2017; Hall et al., 2017.).

### 3.2 Characteristics of included studies

The meta-analysis was based on 177 studies from 2015 to 2025. The studies included many tens of thousands of people, in sample sizes ranging from 50 to 8,000, mostly from North America and Europe, with a cross-sectional design. The detailed characteristics of each included study are presented in Appendix B.

### 3.3 Risk of bias assessment

All 177 of the included studies were assessed in methodological quality and risk of bias using the relevant JBI critical appraisal tools. As for the results, the overall quality of the evidence base was found to be high. Specifically, 139 studies (78.1%) were assessed as having a low risk of bias, 37 studies (21.3%) were assessed as having a moderate risk of bias, and 1 study (0.6%) was assessed as having a high risk of bias. No studies were eliminated from analysis based on assessment of methodological quality. Individual ratings and assessment for methodological quality for each included study can be found in Appendix C.

### 3.4 Main effect of motivational climate

The random effects meta-analysis included 334 effect sizes from the 177 studies that were included in the analysis, which showed a significant, medium overall effect of motivational climate to youth outcomes. The overall effect size was *r* = 0.31 [95% CI [0.28, 0.34], *p* < 0.001].

Nonetheless, there was also an extreme level of heterogeneity among the studies, indicated by both Cochran's Q-test [Q(333) = 25,221.58, *p* < 0.001] and the *I*^2^ (*I*^2^ = 98.45%). Such evidence of heterogeneity warrants further moderator analyses to investigate sources of this variance.

### 3.5 Analysis of moderators

Due to the clear heterogeneity observed in the primary analysis, we completed several pre-specified moderator analyses to assess the sources of variance. Specifically, we tested three moderators: (1) type of outcome variable to examine the hierarchical effects model; (2) type of motivational climate to test their relative efficacy; and (3) year of publication to examine any temporal trends.

#### 3.5.1 The moderating effect of outcome variable type: evidence from a hierarchical effects model

To evaluate whether there is a hierarchical path in the influence of motivational climate, we assessed it in sub-group analyses based on the type of outcome variable. The results revealed a clear and statistically significant hierarchical decreasing pattern [QM(4) = 18.44, *p* = 0.001] that provided strong support for our primary hypothesis.

**Figure 2:**
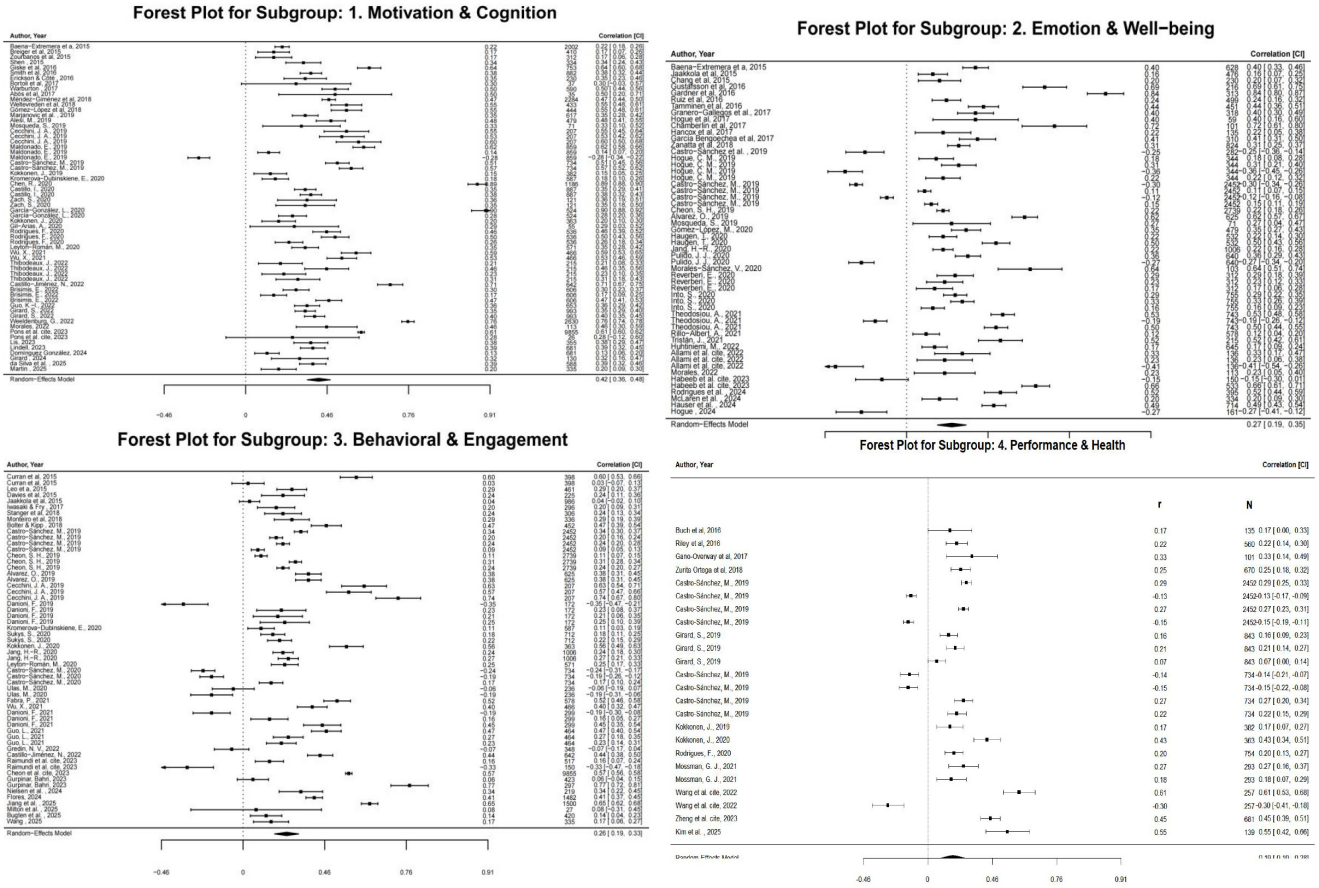
Forest plot.

As shown in Figure 2 and Table 2, the overall effect size for Motivation & Cognition is the highest (*r* = 0.42), and as the effect is transmitted externally, the effect diminishes. The overall effect size for Emotion & Wellbeing is moderate (*r* = 0.27) and even further reduces in effect when this influence applies to Behavioral & Engagement (*r* = 0.26) before the overall effect reaches its weakest level in Performance & Health (*r* = 0.19).

**Table 2:** Subgroup analysis of dependent variables.

**Subgroup (dependent variable)**	**Number of studies**	**Average effect size (*r*)**	**95% confidence intervals (CI)**	**Interpretation of results**
Motivation and cognition	62	0.42	[0.37, 0.52]	High strength
Emotion and Wellbeing	54	0.27	[0.20, 0.34]	Moderate
Behavioral and engagement	58	0.26	[0.19, 0.33]	Medium to weak
Performance and health	24	0.19	[0.07, 0.30]	Weak strength

#### 3.5.2 Subgroup analysis by motivational climate type

A subgroup analysis was conducted to compare the efficacy of different types of motivational climates. The results, detailed in Table 3, indicated that the type of motivational climate was a significant moderator of the overall effect size [QM(df = 4) = 15.93, *p* = 0.0031].

**Table 3:** Subgroup analysis of independent variables.

**Subgroup (independent variable)**	**Average effect size (*r*)**	**95% confidence intervals (CI)**	***p*-value**
Others	0.43	[0.33, 0.42]	<0.001
Autonomy/empowerment/ supportive climate	0.31	[0.23, 0.37]	<0.001
Task/mastery-oriented climate	0.27	[0.22, 0.32]	<0.001
Performance/ego-involved climate	0.17	[0.04, 0.30]	0.0093
Intervention studies	0.22	[0.07, 0.35]	0.0025

As shown in Table 3, four of the five subgroups yielded statistically significant positive effect sizes. The “Others” category, which primarily included “Caring” and “Transformational Leadership” climates, demonstrated the largest pooled effect size at *r* = 0.43 [95% CI [0.33, 0.42]]. This was followed by the “Autonomy/Empowerment/Supportive Climate” [*r* = 0.31, 95% CI [0.23, 0.37]], the “Task/Mastery-Oriented Climate” [*r* = 0.27, 95% CI [0.20, 0.32]]. Notably, both the “Intervention Studies” subgroup [*r* =0 .22, 95% CI [0.07, 0.35]] and the “Performance/Ego-Involved Climate” subgroup [*r* = 0.17, 95% CI [0.04, 0.30]] also produced statistically significant positive effects.

#### 3.5.3 Meta-regression with publication year

**Figure 3:**
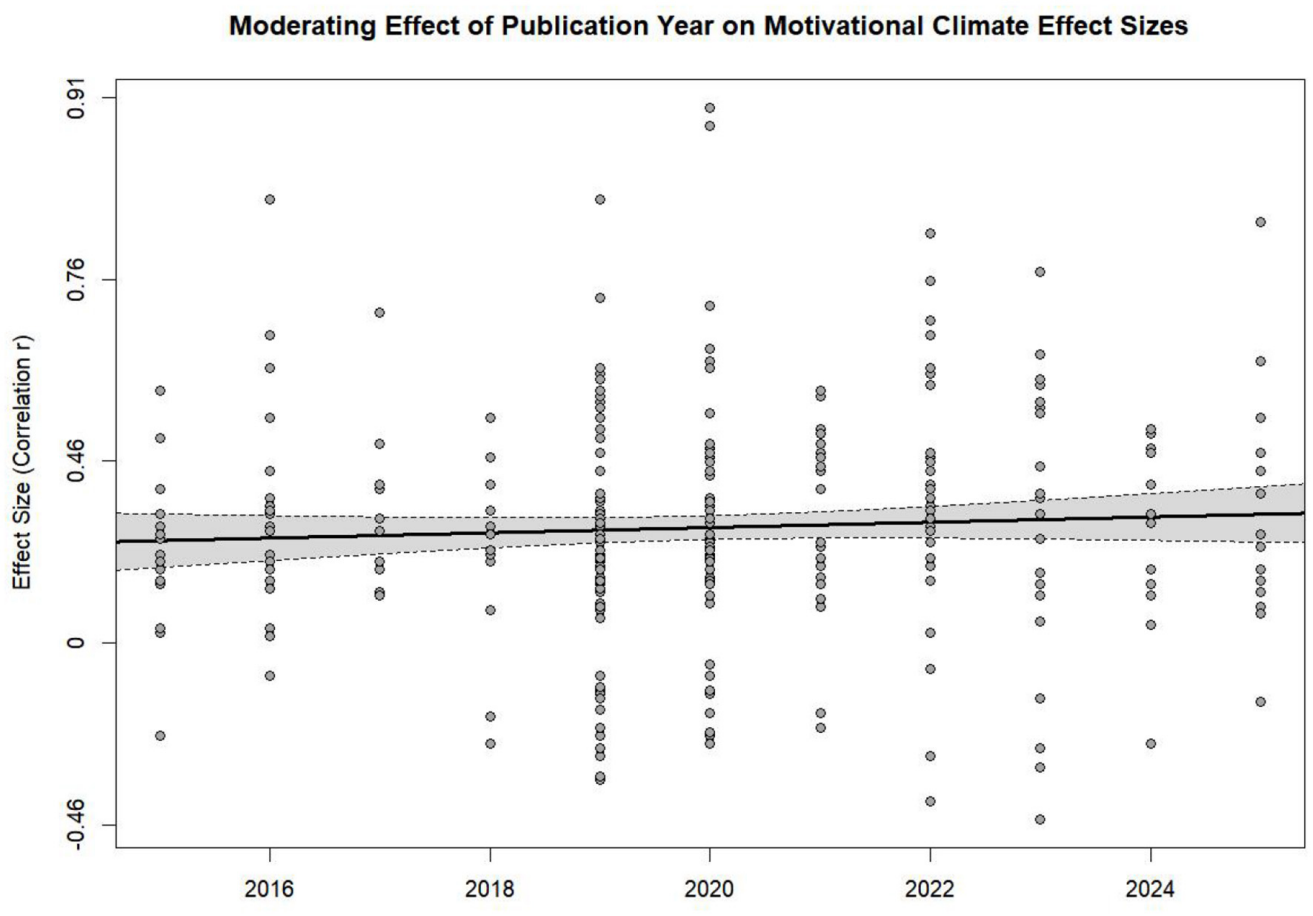
Bubble plot.

To assess the temporal trend of results, a meta-regression was assessed with publication year specified as a moderator. The moderating effect of publication year was not statistically significant [QM(1) = 1.12, *p* = 0.29]. Publication year only reference a minor degree of heterogeneity (*R*^2^ = 0.10%). The bubble plot demonstrating this non-significant trend is shown in Figure 3.

#### 3.5.4 Sensitivity analysis by study design

To further assess the robustness of our findings, a sensitivity analysis was conducted to examine whether the overall effect was moderated by study design. Studies were categorized as Cross-Sectional (k = 313), Experimental (k = 9), or Longitudinal (k = 12). The results of the subgroup analysis indicated that study design was not a statistically significant moderator of the overall effect size [QM(2)=0.23, *p* = 0.89]. The estimated effect sizes for Cross-Sectional (*r* = 0.31), Experimental (*r* = 0.30), and Longitudinal (*r* = 0.27) designs were highly comparable. This finding suggests that the pooled effect of motivational climate is robust and not disproportionately influenced by a specific study design, despite the high heterogeneity present in the overall analysis. When excluding all β-based estimates and analyzing only studies reporting direct correlation coefficients (*r*), the pooled effect size remained highly stable (*r* =0.32). This indicates that the inclusion of converted regression coefficients did not artificially skew the overall results.

The RVE model yielded a pooled effect size of *r* = 0.31 (*p* < 0.001), which is identical to the result from the standard random-effects model. Although the confidence intervals were slightly wider as expected [95% CI [0.26, 0.36]], the statistical significance and magnitude of the effect remained unchanged. This confirms that the hierarchical dependency structure of the data did not compromise the validity of the main conclusions.

### 3.6 Assessment of publication bias

**Figure 4:**
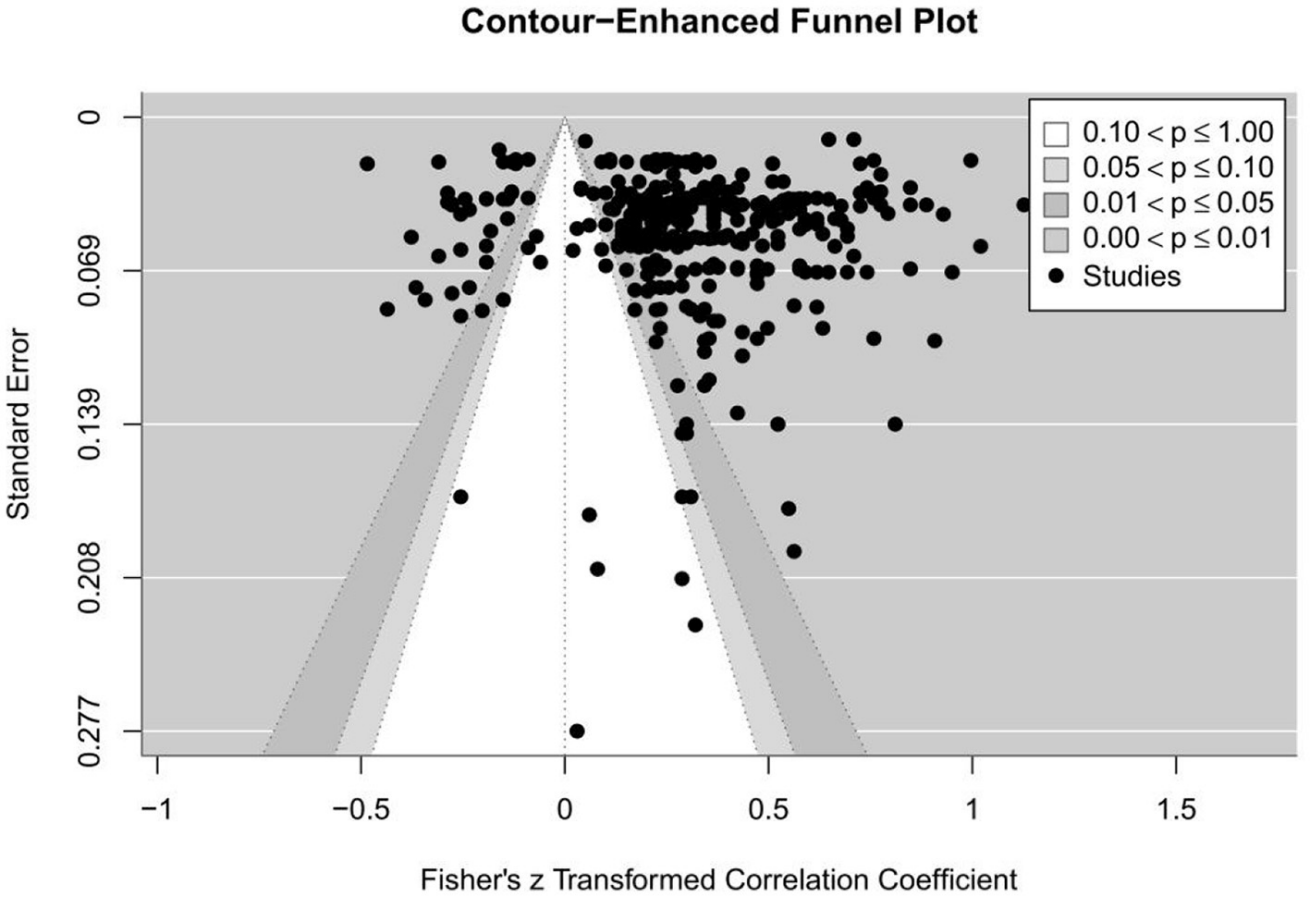
Contour-enhanced funnel plot.

To make a comprehensive examination of publication bias given high heterogeneity (*I*^2^ = 98.45%) involved consideration of several methods. First, a contour-enhanced funnel plot (Figure 4) produced showed symmetrically-distributed studies, consistent with Egger's test for bias (z = 0.11, *p* = 0.91), which showed no significant bias. In addition, a trim-and-fill analysis suggested that zero studies were missing due to publication bias. Taken together, these converging pieces of evidence suggest that publication bias is not likely to be a meaningful concern in this meta-analysis.

### 3.7 Influence diagnostics and robustness check

To determine how robust the pooled estimate was, we conducted a leave-one-out influence analysis. The influence diagnosis results (see Appendix Figure 5 for detailed plots) revealed that no individual study had an excessive influence on the pooled effect size. For each of the studies considered in isolation, the pooled estimate retained stability and the 95% confidence interval never included zero. Overall, these findings suggest that the findings aren't specifically dependent on any one study.

To test the robustness of the main findings again, including only the 139 studies assessed as having a low risk of bias, which comprised 267 effect sizes. The random-effects meta-analysis on this high-quality subset yielded a pooled effect size of *r* = 0.31 [95% CI [0.28, 0.35], *p* < 0.001]. This result is highly consistent with the overall effect size calculated from the full sample (*r* = 0.31).

To further corroborate these findings given the high heterogeneity (*I*^2^ > 98%) which can distort funnel plot symmetry, we conducted a p-curve analysis (Simonsohn et al., 2014). The p-curve included only statistically significant effect sizes (*p* < 0.05) and demonstrated a significantly right-skewed distribution, indicating strong evidential value (Continuous test: Z = −12.4], *p* < 0.001). This suggests that the observed effect is not merely an artifact of selective reporting.

Caveat: Despite these converging lines of evidence (Egger's test, Trim-and-Fill, and p-curve), interpretations regarding publication bias should be made with caution. The extreme heterogeneity observed in this meta-analysis implies that asymmetry in the funnel plot could partially stem from methodological diversity (e.g., small-study effects) rather than bias alone.

## 4 Discussions

The main conclusion from this meta-analysis is the considerable support for a hierarchical influence of motivational climate on youth sport outcomes. Specifically, we observed that climate effects were largest for internal psychological variables (e.g., Motivation and Cognition; *r* = 0.42, Emotion and Wellbeing; *r* = 0.27) and systematically decreased for Behavioral and Engagement (*r* = 0.26), and least for longer-term, distal outcomes like performance and health (*r* = 0.19). Furthermore, we also observed a clear effectiveness hierarchy, such that autonomy-supportive individual and task-orientation climates appeared to have stronger effects than performance-orientation climates. The effect of publication year as a moderator did not appear to be significant.

**Figure 5:**
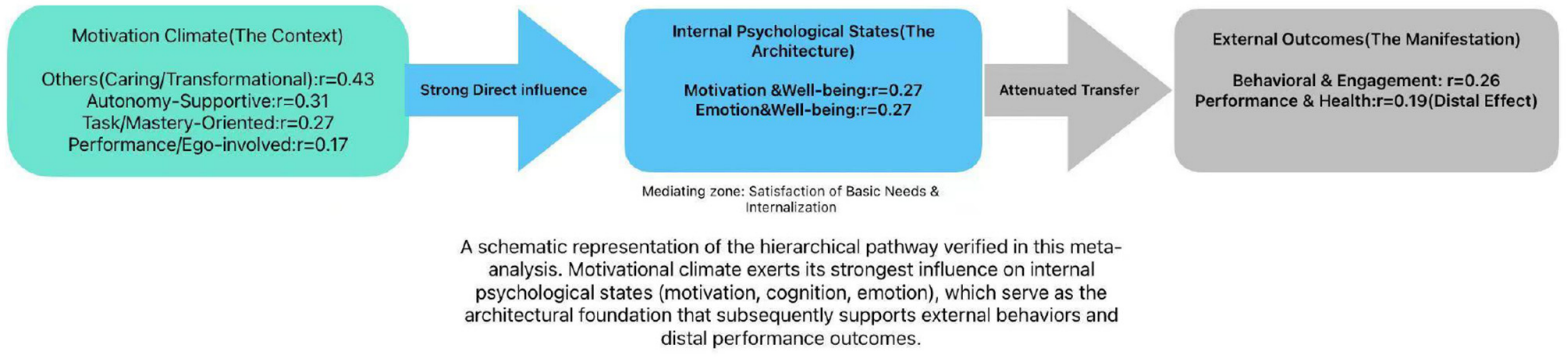
The “psychological architect” model.

The aforementioned pattern of diminishing effect sizes provides a powerful rationale for the inconsistent findings and significant heterogeneity observed in prior studies. The above pattern also strongly suggests that the motivational climate is not a direct determinant of adolescents' external performance. This pattern strongly suggests that motivational climate functions not as a direct determinant of external performance, but as a “psychological architect” (see Figure 5). As illustrated in the conceptual model, the climate first constructs the internal psychological foundation—shaping cognitions and satisfying basic needs—which then translates into observable behavior. This hierarchical finding aligns empirically with the motivational mediation mechanisms proposed in Self-Determination Theory (Ryan and Deci, 2000) 1 and validated in physical education contexts by Standage et al. (2003). These foundational studies posit that social-contextual factors (i.e., climate) do not directly cause behavior; rather, their influence is fully mediated by the satisfaction of basic psychological needs and autonomous motivation. Our meta-analytic results provide large-scale quantitative verification of this theoretical sequence, confirming that the “architectural work” of the climate happens internally before it manifests externally.

### 4.1 Revealing the influence pathway: a hierarchical effect model of motivational climate's action on youth sport outcomes

Moderation analysis constructs a “psychology-behavior-performance” hierarchical effect model of motivational climate's action on youth sport outcomes. The results indicated that the overall effect of motivational climate on “motivation and cognition” (*r* = 0.42) was much larger than its effect on “Emotion and Wellbeing” (*r* = 0.27), followed by its effect on “Behavioral Performance” (*r* = 0.26), and its effect on “Performance and Health” was relatively weak (*r* = 0.19). This order of magnitude gradation in effect sizes shows that the influence of motivational climate is indirect. It influences individuals through shaping individuals' psychological cognitions and further internalizing variables such as motivation and self-efficacy, which in turn cause behavioral transformation and finally lead to performance, rather than influencing individuals through targeting external performance directly. This hierarchical attenuation is not merely statistical but holds profound practical implications. Following recent guidance on interpreting effect sizes in educational practice (Rodríguez-Sabiote et al., 2025), we can quantify this “cascade” in terms of explained variance (*R*^2^).

Psychological Level (*r* = 0.42, *R*^2^≈18%): A positive climate explains a substantial portion of students' internal states. In practice, this means that when a teacher shifts from a controlling to an autonomy-supportive style, the most immediate and visible impact is on students' confidence and enjoyment.

Behavioral Level (*r* = 0.26, R^2^≈7%): The influence on actual engagement (e.g., attendance, intensity) is moderate. This suggests that while climate fuels the “will” to participate, other constraints (e.g., skill level, logistics) also play a role.

Performance Level (*r* = 0.19, R^2^≈4%): The impact on objective performance (e.g., winning, fitness scores) is the weakest.

The finding is highly consistent with the internal logic of Self-Determination Theory (SDT) and Achievement Goal Theory (AGT). These two theories hold that the environment first acts on individuals' basic psychological needs and cognitive appraisals (perceived competence, goal orientation), and the changes in individuals' internal psychological states are the basis for subsequent changes in emotion, behavior, and finally, performance. The hierarchical effect model supports both theories. That is, motivational climate is like an “architect of psychology” rather than an “enhancer of performance” in sports activities. In the future, physical educators should focus on assessing psychological indicators such as students' motivation, confidence, and wellbeing when evaluating the effectiveness of their teaching climate, because these indicators are the basis for achieving long-term positive behavioral outcomes.

### 4.2 The hierarchy of efficacy: the relative importance of the types of motivational climate

This meta-analysis also establishes a clear efficacy hierarchy among different types of motivational climates. The results indicated that the type of motivational climate was a significant moderator of the overall effect size [QM(4) = 15.93, *p* = 0.0031]. Consistent with foundational theories, climates emphasizing autonomy and personal mastery yielded larger positive effects than those centered on social comparison. Specifically, “Autonomy/Supportive” (*r* = 0.31) and “Task/Mastery” (*r* = 0.27) climates produced stronger associations with positive outcomes than the “Performance/Ego” climate (*r* = 0.17). This provides robust, large-scale evidence that practitioners should focus on fostering an atmosphere of self-improvement and empowerment over one based heavily on normative success.

A finding of particular interest was the robust effect size associated with the “Others” classification (*r* = 0.43), which largely consisted of “Caring” and “Transformational Leadership” climates. Despite being less frequently studied than the traditional Task/Ego dichotomy, their superior efficacy suggests they may offer a more holistic support system for youth. The potency of a “Caring Climate” likely stems from its unique capacity to satisfy the need for relatedness. unlike standard task-oriented climates that focus on skill mastery, a caring climate emphasizes interpersonal safety, mutual respect, and the sense of being valued as a person rather than just an athlete. This deep sense of belonging reduces social anxiety and fear of failure, creating a secure psychological base that fosters intrinsic motivation. Similarly, “Transformational Leadership” appears to exert a “multi-need” effect. By offering “inspirational motivation” and “individualized consideration,” this climate not only clarifies the path to success (enhancing competence) but also aligns team goals with personal values (supporting autonomy). Consequently, these relational and inspirational models arguably provide a more comprehensive “nutrient” profile for adolescent psychological needs than climates that focus solely on task structure.

Interestingly and contrary to some preconceptions, the “Intervention Studies” sub-category also produced a statistically significant positive effect (*r* =0.22; *p* = 0.0025). This finding is promising in that it indicates that a structured program that aims to alter the motivational climate in youth sport settings can have a meaningful positive effect despite the methodological cumbersomeness of the interrupted case-study designs (e.g., timing of week's assignment, fidelity of instructor's implementation, limited sensitivity of instruments).

### 4.3 The moderating role of publication year

While a non-significant finding is important to note, this finding implies that there had not been simple linear trend in the relationship of motivational climate and positive outcomes over the past 10 years. More importantly, this finding suggests that the large heterogeneity in this research area is not simply explained by methodological advances or trends over time. Conversely, the findings of this meta-regression suggest that the substantive theoretical moderators, such as the hierarchical nature of the outcomes and the specific type of climate, are the main contributors to the observed variance in the literature. Thus, these findings suggest that the general findings in this area of research are relatively stable, and to understand differences across studies, one must look to deeper theoretical meanings rather than simply holding the publication year constant.

### 4.4 From consensus to practice: the “silent stage” of pedagogy

The findings offer a resolution to the historical lack of consensus regarding climate interventions. Previous inconsistencies may have stemmed from measuring outcomes at different levels of the hierarchy without accounting for the “psychological lag.” The hierarchical model suggests that the effects of a positive climate are not immediately visible in behavior. For practitioners and PE teachers, this implies a critical shift in expectation: The absence of immediate behavioral improvement (e.g., higher physical activity levels) does not indicate intervention failure. Instead, educators should focus on the “Silent Stage”—cultivating the internal psychological architecture (motivation and wellbeing). Once these internal bases are solidified, behavioral engagement is likely to follow as a natural downstream consequence. Therefore, assessment of teaching effectiveness should prioritize psychological indicators over immediate performance metrics.

To operationalize these findings, professional development for coaches and teachers must evolve beyond technical instruction. Recent empirical evidence confirms that targeted SDT-based training programs can successfully equip educators with the specific behavioral strategies needed to foster inclusive, autonomy-supportive climates (Ocete et al., 2025). Furthermore, training should explicitly educate practitioners on the motivational mechanisms at play; understanding how teaching styles mediate basic psychological needs is crucial for maximizing their impact on students' academic self-concept and intrinsic motivation (Espinoza Gutiérrez et al., 2024).

### 4.5 Differences in situations under different roles

Climate effects may vary by context. Analysis revealed that the “Others” category—which heavily features Caring and Peer-initiated climates—yielded the strongest effects (*r* = 0.43). This challenges the traditional “Coach-Centric” model of sports motivation. Contextually, this suggests a functional differentiation: Coaches & Teachers (Structured Context): Often focus on Task/Mastery climates (*r* = 0.27) to build competence and skill structure. Peers & Social Environment (Relational Context): Likely drive the Caring/Transformational effects (*r* = 0.43) by satisfying the need for relatedness and emotional safety. This implies that a “Task-Oriented” class alone is insufficient. A truly effective ecosystem requires integrating the structural guidance of teachers with the relational warmth of a caring peer climate. Future interventions should thus move beyond the “teacher-student” dyad to engineer the broader social network of the youth athlete.

### 4.6 Limitations and future directions

The main limitation of this meta-analysis is extreme heterogeneity (*I*^2^ > 98%) combined with a standard random-effects model that omits the dependent structure of the effect sizes. Many of the effect sizes included above are from the same primary studies (i.e., multiple outcomes taken from the same sample). We treat these effect sizes in the analysis as independent, however, this is a poor model and can lead to estimates of the pooled estimates that are biased and confidence intervals that are underestimated. Future studies should strive to improve upon this limitation by using a more sophisticated model, like multilevel meta-analysis or robust variance estimation (RVE), that considers dependent effect sizes.

Secondly, although the re-categorization of motivational climates and outcome variables was necessary in this analysis, it is still an unavoidable simplification. For instance, the broader “Others” category represented a number of specific climate types and outcomes that could not have effect sizes calculated or were too diverse to synthesize. Future research could benefit from more primary studies that consider the nuances around these constructs, for instance, around the “caring” climate so we can undertake more specific meta-analytic inquiry.

Although the “Others” category (Caring/Transformational) demonstrated the strongest association with positive outcomes (*r* = 0.43), interpretations should be tempered by the relative scarcity of empirical data. Specifically, this subgroup represented a smaller portion of the total pool (k = 25) compared to the extensive literature on Task/Mastery-Oriented climates (k = 94). Furthermore, existing studies on these emerging climates are predominantly cross-sectional and concentrated in Western educational contexts. This geographical and methodological homogeneity limits our ability to generalize their efficacy across diverse cultural settings or to assert causal directionality. Therefore, a critical gap remains: Future scholarship must move beyond correlational validation and employ longitudinal or intervention-based designs to rigorously test whether Caring and Transformational climates can be systematically implemented to enhance youth engagement over time.

Lastly, it is crucial to interpret the hierarchical model as a pattern of association rather than a confirmed causal mechanism. Therefore, future research should strive to use longitudinal and experimental high quality designs to better explicitly foreground the long-term causative roles of motivational climate. Such studies would also be important to contribute even more effective intervention action and high degree of replication to stimulate more theoretical understanding and theory into practice in physical education lesson contexts.

## 5 Conclusion

This meta-analysis confirms a consistent positive association between motivational climate and positive youth sport outcomes. The primary contribution of this study is in providing strong support for a psychology-behavior-performance hierarchical effect model. This model indicates that the climate's association with positive outcomes is graded, beginning with a strong association with internal psychological states that attenuates for external behaviors and performance. This finding positions the motivational climate as a crucial “psychological architect” rather than a direct performance enhancer. In addition, the results provide a clear ordering of the strength of associations, with autonomy-supportive and task-oriented climates associating stronger with positive outcomes than performance-oriented climates and highlighting the important, if often overlooked opportunities for caring and transformational leadership climates. This adds further clarity to both the theoretical implications and the degree of certainty to evidence-informed recommendations for practice for those practitioners working with young people in sport and physical education for positive development.

## Data Availability

The original contributions presented in the study are included in the article/Supplementary material, further inquiries can be directed to the corresponding author.
