# Supplementary material for: A systematic review and meta-analysis of motivational climate and youth sport outcomes: examining a hierarchical effects model
Source: Front Psychol. 2026 Jan 8;16:1716745. doi: 10.3389/fpsyg.2025.1716745 (PMC12823993; doi:10.3389/fpsyg.2025.1716745)
Supplement: Supplementary file 1 [file Supplementary_file_1.docx]

TS=("motivational climate" OR "motivational environment" OR "achievement climate" OR "empowering climate" OR "disempowering climate" OR "caring climate" OR "mastery climate" OR "performance climate" OR "task-involving climate" OR "ego-involving climate")

AND

TS=("physical education" OR PE OR sport* OR "physical activity" OR exercise OR athlete* OR student* OR coach* OR teacher* OR player* OR team* OR "physical domain")

AND

TS=(outcome* OR effect* OR impact* OR consequence* OR correlate* OR predict* OR antecedents OR

motivation* OR engagement OR participation OR persistence OR dropout OR adherence OR

behavio* OR skill* OR performance OR

emotion* OR affect* OR anxiety OR enjoyment OR satisfaction OR

cognit* OR perception* OR "self-efficacy" OR "self-esteem" OR "goal orientation*" OR "self-determination" OR

"psychological well-being" OR "mental health" OR wellbeing OR stress OR burnout)

https://webofscience.clarivate.cn/wos/alldb/summary/33eacae8-3e40-4ce8-bede-3b4995a697ea-016ccccb14/relevance/1
