# Supplementary figures and images for: A systematic review and meta-analysis of motivational climate and youth sport outcomes: examining a hierarchical effects model
Source: Front Psychol. 2026 Jan 8;16:1716745. doi: 10.3389/fpsyg.2025.1716745 (PMC12823993; doi:10.3389/fpsyg.2025.1716745)

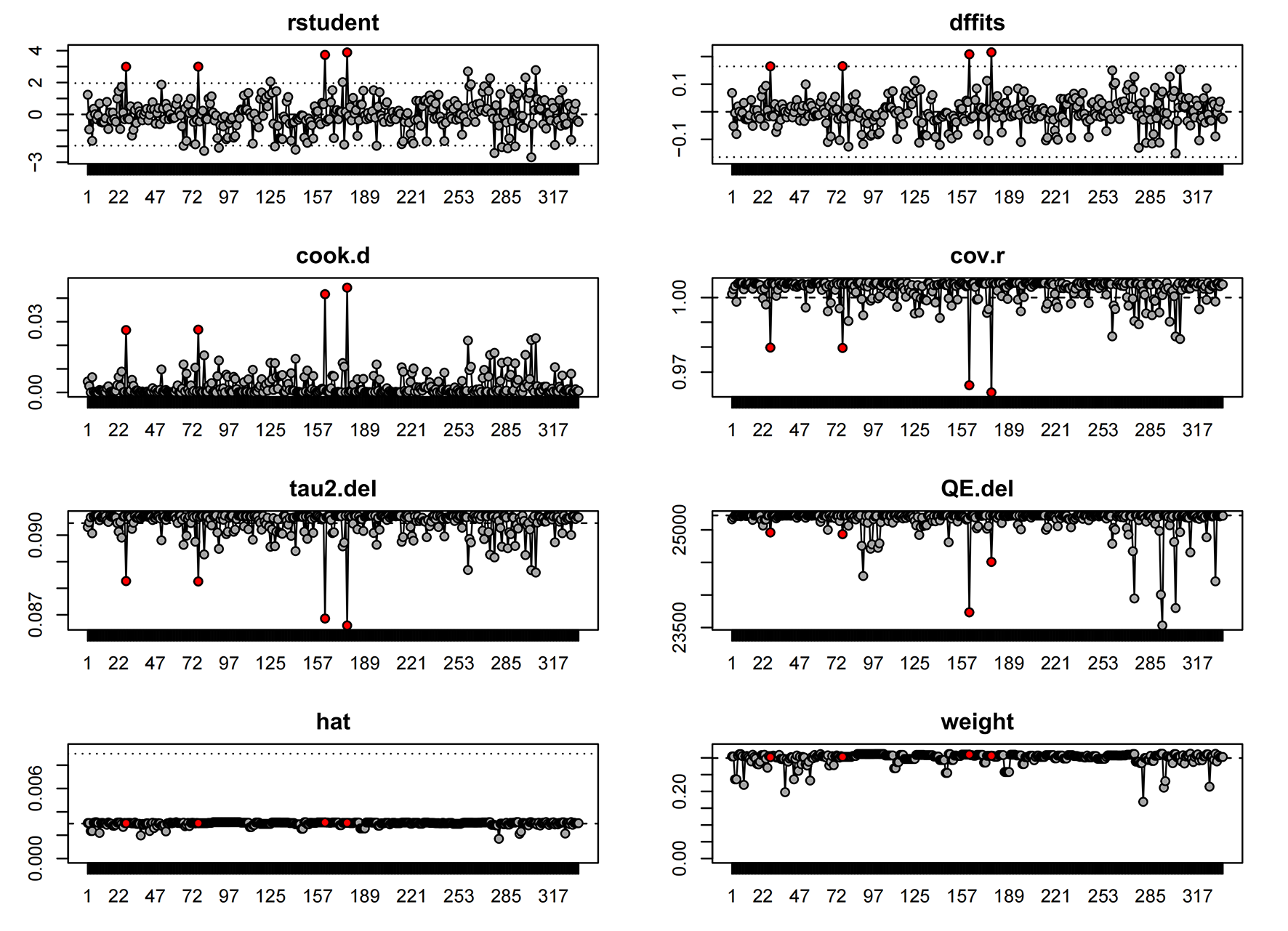

Supplement: Supplementary file 4 [file Image_5.png]
